# Improving the Production of Riboflavin by Introducing a Mutant Ribulose 5-Phosphate 3-Epimerase Gene in *Bacillus subtilis*

**DOI:** 10.3389/fbioe.2021.704650

**Published:** 2021-07-29

**Authors:** Bin Yang, Yiwen Sun, Shouying Fu, Miaomiao Xia, Yuan Su, Chuan Liu, Chunzhi Zhang, Dawei Zhang

**Affiliations:** ^1^School of Biological Engineering, Dalian Polytechnic University, Dalian, China; ^2^Tianjin Institute of Industrial Biotechnology, Chinese Academy of Sciences, Tianjin, China; ^3^College of Biotechnology, Tianjin University of Science and Technology, Tianjin, China; ^4^Key Laboratory of Systems Microbial Biotechnology, Chinese Academy of Sciences, Tianjin, China; ^5^University of Chinese Academy of Sciences, Beijing, China

**Keywords:** riboflavin, ribulose 5-phosphate 3-epimerase, pentose phosphate pathway, truncated protein, *Bacillus subtilis*

## Abstract

Ribulose 5-phosphate (Ru5P) and guanosine 5′-triphosphate (GTP) are two key precursors of riboflavin, whereby Ru5P is also a precursor of GTP. Ribulose 5-phosphate 3-epimerase (Rpe) catalyzes the conversion of ribulose 5-phosphate into xylulose 5-phosphate. Inactivation of Rpe can reduce the consumption of Ru5P, enhancing the carbon flux toward riboflavin biosynthesis. Here we investigated the effect of mutation of *rpe* and other related genes on riboflavin production, physiological and metabolic phenotypes in *Bacillus subtilis* LY (BSLY). Introducing single nucleotide deletion (generated BSR) or nonsense mutation (generated BSRN) on the genomic copy of *rpe*, resulting in more than fivefold increase of riboflavin production over the parental strain. BSR process 62% Rpe activity, while BSRN lost the entire Rpe activity and had a growth defect compared with the parent strain. BSR and BSRN exhibited increases of the inosine and guanine titers, in addition, BSRN exhibited an increase of inosine 5′-monophosphate titer in fermentation. The transcription levels of most oxidative pentose phosphate pathway and purine synthesis genes were unchanged in BSR, except for the levels of *zwf* and *ndk*, which were higher than in BSLY. The production of riboflavin was increased to 479.90 ± 33.21 mg/L when *ribA* was overexpressed in BSR. The overexpression of *zwf*, *gntZ*, *prs*, and *purF* also enhanced the riboflavin production. Finally, overexpression of the *rib* operon by the pMX45 plasmid and mutant *gnd* by pHP03 plasmid in BSR led to a 3.05-fold increase of the riboflavin production (977.29 ± 63.44 mg/L), showing the potential for further engineering of this strain.

## Introduction

Riboflavin, also known as Vitamin B_2_, is an isoalloxazine derivative that was previously produced by chemical synthesis. But over the last decades, microbial fermentation has become dominant in its industrial production (Schwechheimer et al., 2016). Animals must acquire this essential cofactor from food sources (Pinto and Zempleni, 2016), while most of bacteria, fungi, and plants can synthesize it using native metabolic pathways. The most commonly used riboflavin producers are bacteria and fungi, such as *Bacillus subtilis* (Zhu et al., 2006; Shi et al., 2009; Wang et al., 2018, 2019; Liu et al., 2020), *Ashbya gossypii* (Aguiar et al., 2015; Schwechheimer et al., 2018), and *Candida famata* (Dmytruk et al., 2011). As a traditional industrial strain, *A. gossypii* can produce 15 g/L riboflavin in fed-batch fermentation (Stahmann et al., 2000), while *Candida famata* can produce 20 g/L riboflavin after 200 h of fermentation (Heefner et al., 1994). *B. subtilis* is a superior host, which can reach a titer of 20–27 g/L, after a rapid fermentation process of 3 days (Lee et al., 2008).

**FIGURE 1:**
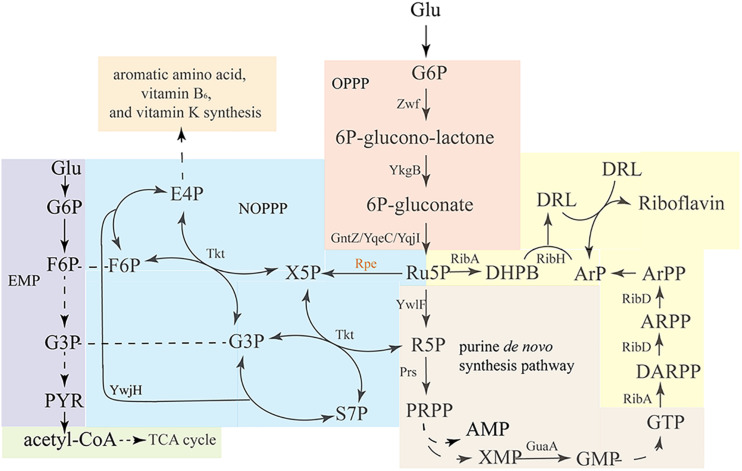
Overview of the riboflavin biosynthesis pathway and related pathways in *B. subtilis*. Glucose enters the oxidative pentose-phosphate pathway (OPPP, orange), and is converted into Ru5P, which enters the purine *de novo* synthesis pathway (brown), which generates GTP. Ribulose-5-phosphate-3-epimerase catalyzes the interconversion of Ru5P and X5P, enabling the conversion of pentose phosphates back into F6P and G3P via transketolase and transaldolase reactions. EMP (purple) and OPPP are linked via the non-oxidative PPP (NOPPP, blue). As the precursors, GTP and Ru5P enter the riboflavin biosynthesis pathway (yellow) to generate riboflavin. Zwf, glucose 6-phosphate dehydrogenase; YkgB, 6-phosphogluconolactonase; GntZ/YqeC/YqjI, 6-phosphogluconate dehydrogenase; YwlF, ribose-5-phosphate isomerase; Prs, phosphoribosylpyrophosphate synthetase; Rpe, ribulose 5-phosphate 3-epimerase; Tkt, transketolase; YwjH, transaldolase; GuaA, GMP synthetase; RibA, GTP cyclohydrolase II/3,4-dihydroxy-2-butanone 4-phosphate synthase; RibD, 5-amino-6-(5-phosphoribosylamino)uracil reductase; RibE, riboflavin synthase (alpha subunit); RibD, riboflavin synthase (beta subunit).

The riboflavin biosynthesis pathway and its regulation in *B. subtilis* have been comprehensively studied (Bacher et al., 1999; Adelbert et al., 2001; Schwechheimer et al., 2016). The two precursors ribulose 5-phosphate (Ru5P) and guanosine 5′-triphosphate (GTP) are derived from the oxidative pentose phosphate pathway (OPPP) (Figure 1 orange area) and purine *de novo* biosynthesis pathway (Figure 1 brown area), respectively. Then, Ru5P and GTP enter the riboflavin synthesis pathway (Figure 1 yellow area), which is composed of a series of enzymes encode by the *rib* operon. As a branch of central carbon metabolism, the pentose phosphate pathway (PPP) generate of pentoses from hexoses through oxidative reactions, and at the same time produces NADPH, which serves as the major source of reducing power for biosynthetic reactions (Figure 1 orange area) (Lehninger et al., 1993). Ru5P is the direct product of the OPPP, and it converted into ribose 5-phosphate (R5P) by ribose 5-phosphate isomerase. Then, R5P is converted into phosphoribosylpyrophosphate (PRPP) by phosphoribosylpyrophosphate synthetase. PRPP is a precursor for the *de novo* biosynthesis of L-histidine, L-tryptophan, nicotinamide adenine dinucleotide^+^ (NAD^+^), nicotinamide adenine dinucleotide phosphate^+^ (NADP^+^), pyrimidines, and purines, including GTP, the other precursor for riboflavin biosynthesis.

Many regulatory mechanisms of riboflavin biosynthesis have been studied. The 5′-untranslated region of the mRNA of the *rib* operon contains a riboswitch sequence, which can form a stem-loop structure when bound to flavin mononucleotide (FMN), causing premature transcription termination (Winkler et al., 2002). Similar to the *rib* operon, the purine operon also contains a riboswitch region that can bind purine compounds to form a secondary structure (Maumita et al., 2003). The transcriptional regulator PurR controls the expression of the purine operon at the transcriptional level (Saxild et al., 2001). Additionally, the purine *de novo* synthesis pathway is regulated through feedback inhibition of enzymes. For example, phosphoribosyl-pyrophosphate synthetase (Prs) is inhibited by ADP and GDP (Arnvig et al., 1990; Eriksen et al., 2000), while glutamine phosphoribosylpyrophosphate amidotransferase (PurF) is inhibited by ATP, ADP, AMP, GTP, GDP, and GMP (Smith et al., 1994). The enzymes in the PPP are also regulated by metabolic intermediates and products of the pathway. For example, glucose-6-phosphate dehydrogenase (G6PD) and 6-phosphogluconate dehydrogenase (6GPD) are inhibited by reduced form of nicotinamide adenine dinucleotide phosphate (NADPH), ATP, and fructose 1,6-bisphosphate (FBP) (Ohnishi et al., 2005).

Many metabolic engineering strategies were developed to modify the flux of the synthetic pathways and regulatory elements in riboflavin-producing strains. To enhance the biosynthesis of Ru5P, G6PD was overexpressed in *B. subtilis* PY, which led to a 25% increase of riboflavin production (Duan et al., 2010). Feedback-resistant mutants of G6PD and 6GPD from *Corynebacterium glutamicum* were also characterized, and applied to improve the production of riboflavin in *B. subtilis* (Wang et al., 2011). The transcriptional regulator gene *purR* and riboswitch sequence were deleted, which deregulated the purine *de novo* synthesis pathway to improve the supply of GTP (Shi et al., 2014).

As discussed above, Ru5P is a precursor for both riboflavin and GTP synthesis, and enhancing its synthesis or decreasing its consumption could have twofold benefits for riboflavin production. Produced by the OPPP (Figure 1 orange area), Ru5P can enter the non-oxidative pentose phosphate pathway (NOPPP) (Figure 1 blue area) to generate other sugar phosphates, such as erythrose 4-phosphate (E4P), which is a precursor for the biosynthesis of aromatic amino acids, vitamin B6, and vitamin K (Stincone et al., 2015). Ribulose 5-phosphate 3-epimerase (encoded by *rpe* in *B. subtilis*) catalyzes the first step of the NOPPP, converting Ru5P into xylulose 5-phosphate (X5P). In previous studies, *B. subtilis* strains possessing a mutant *rpe* or *tkt* gene were found to produce D-ribose (Ken-ichi and Masahiko, 1974; Wulf and Vandamme, 1997), shown a potential for riboflavin overproduction. However, these articles did not specify the sequence of the relevant mutation. While other study discussed possible links between the transcription lever of *rpe* and riboflavin production (Tnnler et al., 2008), the influence of the mutation or deletion of *rpe* on riboflavin production has not been studied yet. In this study, the chromosomal *rpe* gene of a riboflavin-producing *B. subtilis* was mutated by homologous recombination. Then, the riboflavin production, physiology, and metabolic characteristics of the resulting engineered strain were studied. Additionally, other genes were overexpressed in this strain to further enhance riboflavin production. This work exemplifies a strategy for improving the riboflavin production in *B. subtilis* by mutating the *rpe* gene, and may serve as a reference for detecting potential bottlenecks in riboflavin biosynthesis in *rpe* mutant strains.

## Results

### Mutation of Ribulose 5-Phosphate 3-Epimerase Dramatically Increases the Production of Riboflavin in *B. subtilis*

**FIGURE 2:**
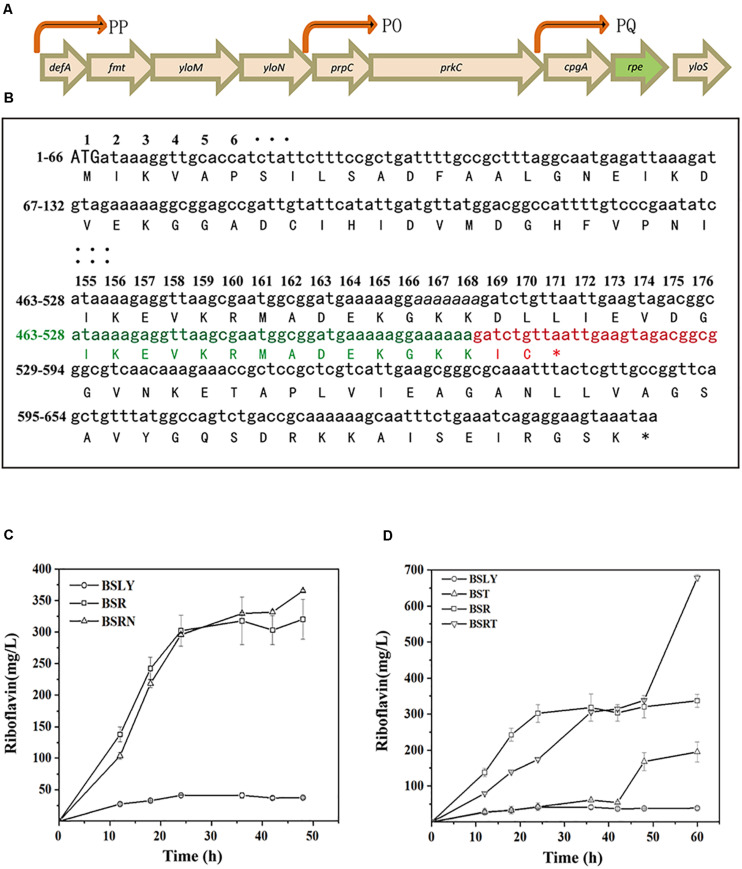
Location and sequence of the *rpe* gene, and riboflavin productivity of *rpe* mutants. **(A)** Location of *rpe* and other eight genes of the operon on the genome. The arrows were indicated promoters (PP, PQ, and PO) confirmed in previous studies (Iwanicki et al., 2005; Hinc et al., 2006). **(B)** Nucleotides and amino acids sequence of the wild-type and mutant *rpe*. The numbers on the left indicate the start and stop nucleotide numbers of each line; the numbers on top are the amino acid numbers of the corresponding codons. Sequence of the wild-type *rpe* is shown in black, and adenines from positions 498 to 504 are shown in italics. Sequence of the mutant *rpe* gene is shown in green (identical with WT sequence) and red (shifted sequence), while the changed amino acids sequence of Rpe is also shown in red. **(C)** Comparison of riboflavin production between BSR (*rpe*504delA), BSRN (*rpe*^0^), and their parent strain. **(D)** Comparison of riboflavin production between BST (*tkt*^0^), BSRT (*rpe*504delA, Δ*tkt*^0^), and their parent strain.

In the first step of NOPPP, ribulose 5-phosphate 3-epimerase converts Ru5P into X5P, so that the pentose phosphate can flow back into the Embden-Meyerhof-Parnas-pathway (EMP). The chemical structure of X5P allows transketolase (Tkt) to transfer a two-carbon glycoaldehyde unit to R5P, generating sedoheptulose 7-phosphate (S7P) and glyceraldehyde 3-phosphate (G3P). Furthermore, transketolase can generate fructose 6-phosphate (F6P) and erythrose 4-phosphate (E4P) from S7P and G3P. In *B. subtilis*, ribulose 5-phosphate 3-epimerase is encoded by *rpe*, which is part of a putative operon with nine genes (Tnnler et al., 2008; Figure 2A, including *defA*, *fmt*, *yloM*, *yloN*, *prpC*, *prkC*, *cpgA*, *rpe*, and *yloS*). None of the other eight genes has a known function related to riboflavin biosynthesis. In 2015, the genome of an EU-unauthorized genetically modified riboflavin-producing *B. subtilis* strain was sequenced (Elodie et al., 2015), and we found an *rpe* mutation in the genomic database of this strain. One of seven adenines from position 498 to 504 is missing in the nucleotide sequence of this mutant, which shifts the reading frame, resulting in a truncated Rpe protein (217–170 aa) (Figure 2B).

To clarify the influence of the *rpe* mutation on riboflavin production, a riboflavin producer *B. subtilis* LY (BSLY), was constructed on the background of *B. subtilis* 168 according to previous references. BSLY carries a deregulated *rib* operon on the chromosome (Shi et al., 2014), as well as a mutation in the adenylosuccinate synthetase [PurA (P242L)] (Wang et al., 2018) that reduces the carbon flow from IMP to AMP. After 48 h of fermentation with sucrose as the carbon source, BSLY produced 37.29 ± 1.32 mg/L of riboflavin. The mutant *rpe* gene was introduced into the wild-type chromosomal *rpe* locus of BSLY to generate *B. subtilis* R (BSR), which produced remarkably more riboflavin (320.50 ± 31.28 mg/L) compared to BSLY (Figure 2C), although its growth rate was insignificantly reduced (Supplementary Figure 2). To clarify whether the increase of production was caused by decreased Rpe activity, we introduced a nonsense *rpe* mutation (the start codon ATG was mutated into the stop codon TAA) at the same locus to generate BSRN. BSRN produced 365.69 ± 27.53 mg/L of riboflavin, while the mutation significantly inhibited the growth of the strain (Supplementary Figure 2). It indicated that the truncation of Rpe may to a certain extent decrease the activity of this enzyme, and thereby lead to a decrease of the carbon flow back to the EMP via the NOPPP.

As key enzymes in NOPPP, the deficiencies of Rpe and Tkt were previously reported to increase the production of D-ribose in *B. subtilis* (Wulf and Vandamme, 1997; Wu et al., 2009). To test the potential of *tkt* mutants in riboflavin production, we therefore introduced a nonsense mutation of *tkt* (the start codon ATG was mutated into the stop codon TAA) in the BSLY and BSR to generate BST and BSRT, respectively. These mutations led to an improvement of riboflavin production in shake-flasks fermentation after 48 h. The riboflavin titer of BST reached 194.09 ± 27.76 mg/L at 60 h (Figure 2D), which was a fivefold increase over the parental strain. Moreover, the riboflavin titer of BSRT reached 678.53 ± 8.81 mg/L at 60 h (Figure 2D), twofold higher than that of BSR. And the growth of these mutants was hampered compared to the parent strains (Supplementary Figure 2). These results indicate that the inactivation of Tkt aggravated the deficiency of the cells to reuse pentose phosphates, and the blockage of the NOPPP remarkably increased the carbon flow toward riboflavin biosynthesis.

### Growth Phenotype and Enzyme Activity of Mutants Deficient in Rpe

**FIGURE 3:**
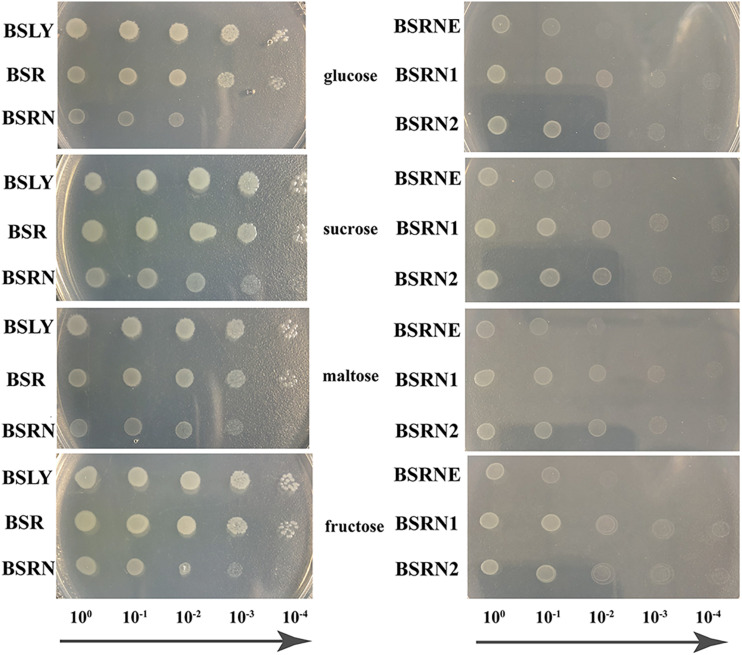
Growth phenotypes of the wild-type and mutant strains grown in GMI media with glucose/sucrose/maltose/fructose as carbon source. **(Left)** BSLY with wild-type *rpe*, BSR carrying mutant *rpe*, BSRN carrying a nonsense mutation of *rpe*. **(Right)** BSRNE is a derivative of BSRN harboring the empty vector pHP13(spe), BSRN1 harboring pHP01 carrying the mutant *rpe*, BSRN2 harboring pHP02 carrying wild-type *rpe*.

The mutation of *rpe* led to a growth defect, which was shown in fermentation. To evaluate this deficiency more systematically and accurately, GMI media with four different carbon sources (glucose, sucrose, maltose, and fructose) were used in the growth experiments. Overnight cultures of BSLY, BSR, and BSRN in LB were washed with GMI medium contained the indicated carbon source and cultured on plates. The growth of BSR was insignificantly slower than that of BSLY, while BSRN showed a marked growth defect (Figure 3 left). The expression of wild-type and truncated Rpe restored the growth of BSRN (Figure 3 right) which indicated that Rpe catalyzes a reaction that provides a precursor needed for rapid biosynthesis of cellular components. We inferred that this metabolite is E4P, which is a precursor of aromatic amino acids, vitamin B_6_, and vitamin K. Although deficient in Rpe, E4P can also be generated by transketolase using glyceraldehyde 3-phosphate and fructose 6-phosphate as substrates. To confirm the metabolic defect in the *rpe* mutants, the cell extracts of BSLY, BSR, and BSRN were used to test their Rpe activities. The conversion rate of Ru5P to X5P in BSLY was 0.707 ± 0.115 μmol/min/μg protein compared to 0.436 ± 0.086 μmol/min/μg protein in BSR. Moreover, there was no detectable Rpe activity in BSRN.

### Investigating the Mechanism of Increased Riboflavin Biosynthesis in the *rpe* Mutant Strain

To investigate the mechanism of riboflavin overproduction in the *rpe* mutants, the purine compounds in the cell culture of BSR, BSRN and BSLY were compared. The cell suspension was extracted at 48 h of the fermentation and the concentrations of purines were measured as section “Materials and Methods” described. As shown in Table 1, the concentrations of AMP and IMP were similar between BSR and BSLY. While the concentrations of GMP and XMP in BSR was 35 and 132% relative to BSLY. And BSR produced 97.31 ± 0.48 μg/L inosine and 71.73 ± 0.65 μg/L guanosine, which was, respectively, 163 and 40%, more than in BSLY. Notably, the titer of IMP of BSRN reached 218.98 ± 1.05 μg/L, which was 6 times that of BSLY. These results indicated that the purine metabolism of these mutants was changed. The increased concentration of IMP may also contribute to the growth defect of BSRN.

**TABLE 1:** Concentration of purine compounds in the cultures of *B. subtilis* strains developed in this study.

Strain	Concentration (μg/L)						
	
	Adenine	Inosine	Guanosine	GMP	AMP	IMP	XMP
BSLY	11.73 ± 0.48	37.07 ± 0.50	50.89 ± 0.82	5.00 ± 0.15	3.07 ± 0.10	6.09 ± 0.16	1.65 ± 0.18
BSR	11.46 ± 0.41	97.31 ± 0.48	71.73 ± 0.65	1.76 ± 0.19	3.05 ± 0.24	6.26 ± 0.19	2.17 ± 0.14
BSRN	16.16 ± 0.68	222.87 ± 1.32	79.38 ± 0.42	3.50 ± 0.39	3.55 ± 0.34	218.98 ± 1.05	1.92 ± 0.21

**FIGURE 4:**
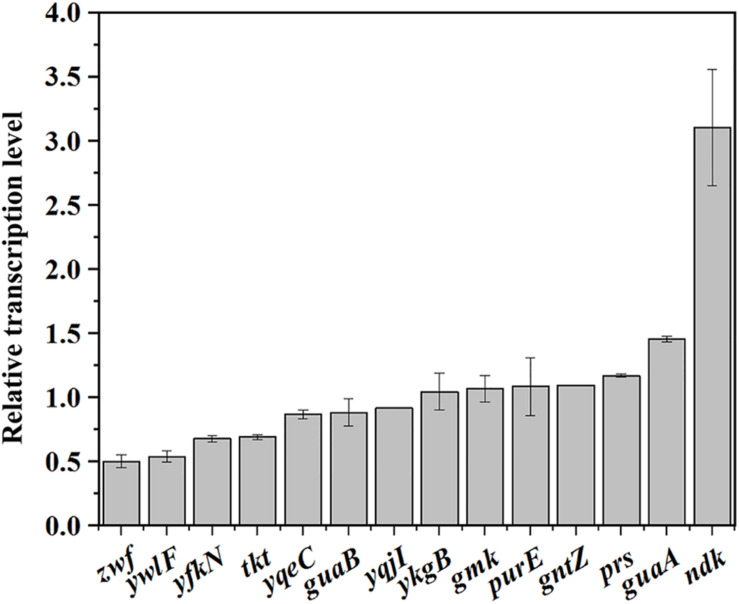
Investigation of the mechanism of riboflavin overproduction in the *rpe* mutant. Relative transcriptional levels of genes in the pathways that provide precursors for riboflavin biosynthesis.

Then, the transcription levels of the genes related to the PPP and purine synthesis pathway in BSR and BSLY were measured by qRT-PCR. As shown in Figure 4B, the transcription levels of these genes were practically the same in BSR and BSLY, except for *zwf* and *ndk*, which, respectively, exhibited a 49% decrease and a 310% increase. These results indicated that the variation of metabolic flux toward purine compounds synthesis did not require a general activation of transcription of the pathway genes. The transcription of the purine operon is regulated by PurR, and its regulation is mediated by adenine (Manli and Howard, 2000). There was also no difference of adenine concentration in these strains (Table 1). Increased expression of *ndk* indicated that there is an imbalance of the nucleotide pools, which was also shown in the results of HPLC analysis (Table 1). In addition, the transcription levels of ribose 5-phosphate isomerase (encoded by *ywlF*), and transketolase (encoded by *tkt*), which also catalyze reactions related to Ru5P, were slightly reduced in the *rpe* mutant (Figure 4).

### Improving the Riboflavin Production by Overexpressing the Genes Related to the PPP, Purine *de novo* Synthesis Pathway and Riboflavin Biosynthesis Pathway

**FIGURE 5:**
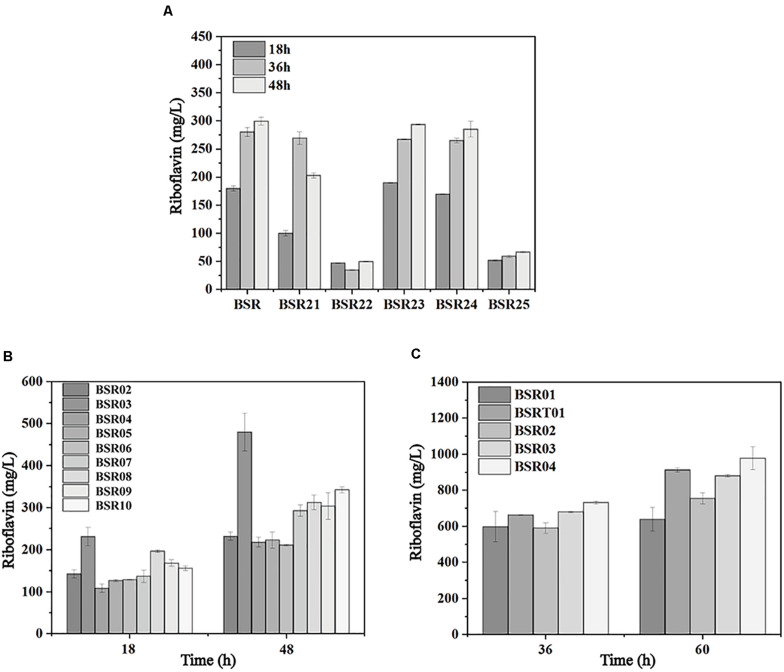
Metabolic engineering of the initial producer strain harboring the *rpe* mutation to further enhance riboflavin production. **(A)** Comparison of riboflavin production in strains individually overexpressing the genes involved in the PPP, purine *de novo* synthesis pathway, and riboflavin biosynthesis pathway. **(B)** Comparison of riboflavin production in strains carrying deletions of genes involved in OPPP and their parent strain. **(C)** Comparison of riboflavin production in strains expressing the *rib* operon.

The sufficient supply of precursors and energy, as well as a proper ratio of the two precursors, is vital for riboflavin biosynthesis. To investigate the limiting reactions in riboflavin synthesis, genes of the PPP, purine *de novo* synthesis pathway, and riboflavin biosynthesis pathway were individually integrated into the mid-copy-number overexpression plasmid pMA5 (spe). These plasmids were introduced into BSR generating BSR02-BSR10. As shown in Figure 5A, with the overexpression of *ribA*, the production of riboflavin reached 479.90 ± 33.21 mg/L at 48 h, representing a 2-fold increase compared with the strain carrying pMA5 (spe). The overexpression of *zwf*, *gntZ*, *purF*, and *prs* increased the production of riboflavin by 48, 31, 26, and 35%, respectively, while the overexpression of *ykgB*, *gmk*, and *guaA* had a negative effect.

These results indicated that the bifunctional enzyme RibA is still a major bottleneck of riboflavin biosynthesis in the *rpe* mutant, which was in agreement with previous studies (Humbelin et al., 1999). Furthermore, the expression of G6PD and 6GPD is beneficial for the supply of precursors, since these enzymes catalyze key steps in the PPP. The expression of *purF* and *prs* increases the flux toward purine biosynthesis, while the expression of *guaA* and *gmk* may cause an accumulation of the intermediates (GMP and GDP) in this pathway, which inhibits the activities of the enzymes of downstream purine biosynthesis. Furthermore deletion of Zwf and YqeC severely impaired the synthesis of riboflavin in the *rpe* mutant (Figure 5B), indicating that these two enzymes are key points that control the flux of OPPP.

To investigate the influence of the expression of the *rib* operon on riboflavin production in the *rpe* mutant, the low-copy-number pSM19035-derived plasmid pMX45 carrying a complete *rib* operon (Wang et al., 2018) was introduced into BSR to generate BSR01. BSR01 produced 637.70 ± 65.69 mg/L riboflavin after 60 h, representing a 90% increase compared with BSR (Figure 5C). This result indicate that the expression of the complete *rib* operon in the *rpe* mutant had a more significant effect on the production of riboflavin. To further enhance the precursor supply, the mid-copy-number plasmid pHP13(spe) carrying the intermediate-strength promoter PvegI was used to express site-directed mutants of G6PD and 6GPD from *C. glutamicum* (Wang et al., 2011). The resulting plasmid pHP03 (G6PD) and pHP04 (6GPD) were introduced into BSR01 to generate BSR03 and BSR04, respectively. The riboflavin titers of BSR03 and BSR04 reached 880.10 ± 7.93 and 977.29 ± 63.44 mg/L at 60 h. On the other hand, the introduction of pMX45 into BSRT generated another strain with high riboflavin production, named BSRT01, which produced 912.68 ± 12.34 mg/L riboflavin at 60 h (Figure 5C).

## Discussion

In this study, a mutant of ribulose 5-phosphate 3-epimerase (*rpe*) was identified in the genome database of a riboflavin producing *B. subtilis* strain. Then, the wild-type *rpe* was mutated in the genome of a *B. subtilis* strain with a clear genetic background to study the effects of the identified mutation on riboflavin production. The results showed that the production of riboflavin was significantly enhanced in the strain carrying the mutant *rpe*. Additionally, we found that the production of riboflavin was also increased when a nonsense mutation of *rpe* replaced the wild-type one. The production of riboflavin was further enhanced in a strain carrying both the mutant *rpe* and a nonsense mutation of *tkt*. We therefore inferred that the overproduction of riboflavin is associated with the accumulation of the precursor Ru5P.

*B. subtilis* is one of the most commonly used ribose producers, reaching titers of up to 27 g/L in batch fermentation, with a yield of 300 mg ribose g^–1^ glucose (Wu et al., 2009). However, the yield of riboflavin in batch fermentation is only 60 mg g^–1^ glucose (Chen et al., 2006). Previous studies of ribose-producing *B. subtilis* strains also showed that many of them carried mutations of *rpe* or *tkt* (Ken-ichi and Masahiko, 1974; Wulf and Vandamme, 1997). Unfortunately, none of these articles and patents reported the sequence of the mutant genes, and it was unclear if the *rpe* mutation in the database (Elodie et al., 2015) is the same as the one in the ribose producing *B. subtilis.* Nonetheless, the enhancement of riboflavin production by the nonsense mutant of *rpe* and the activity data indicated that other mutations that deactivate Rpe might also cause the same phenotype.

The purine metabolism of the *rpe* mutant different from the parent strain, especially for the increased accumulation of inosine and guanosine. However, the transcription levels of genes in the purine *de novo* synthesis pathway and OPPP were not changed, except for *zwf* and *ndk*. It indicated that the variations of the purine metabolism do not require much changes of gene expression at the transcriptional level. Additionally, *B. subtilis* is also a producer of nucleosides such as inosine, adenosine, and guanosine, which were generated by the dephosphorylation of nucleotides (Akio et al., 1965). However, the accumulation of purine compounds can inhibit enzymes in the purine *de novo* synthesis pathway. Thus these compounds must be secreted out of the cell to alleviate inhibition and maintain normal growth.

Wang et al. (2018) combined whole-genome and transcriptome sequence analysis to construct the riboflavin overproduction strain *B. subtilis* BS125. This strain produced 4,232 mg/L riboflavin after 72 h in shake-flasks fermentation with 100 g/L glucose. The parent of the final strain was BS124, in which none of the genes in the NOPPP was changed and the copy number of *rib* operon was increased through plasmid expression and genomic integration, produced 2,525 mg/L of riboflavin. The yield of this strain was 25.25 mg riboflavin g^–1^ glucose, which was comparable to the strains constructed in this study (BSR03: 22.00 mg riboflavin g^–1^ sucrose, BSR04: 24.43 mg riboflavin g^–1^ sucrose, BSRT01: 22.81 mg riboflavin g^–1^ sucrose). In our engineered strains, only the plasmid pMX45 carried additional copies of the *rib* operon. Moreover, we only manipulated BSLY in three steps, to obtain each of these strains, which illustrates the importance and effectiveness of engineering the PPP for riboflavin biosynthesis. To construct a more competitive riboflavin producer, more copies of the *rib* operon are needed to ensure a high carbon flux toward the downstream reactions.

## Materials and Methods

### Strains, Plasmids, and Reagents

The bacterial strains and plasmids used in this study are listed in Supplementary Table 1. All *B. subtilis* strains were derived from the wild-type *B. subtilis* 168. *Escherichia coli* DH5α was used for routine cloning and plasmid propagation. When required, antibiotics were added to the growth media at the following concentrations: 100 μg/mL spectinomycin for *E. coli* selection, 250 μg/mL for *B. subtilis* selection; 20 μg/mL erythromycin for *B. subtilis* selection; 8 μg/mL chloramphenicol for *B. subtilis* selection; 20 μg/mL neomycin for *B. subtilis* selection. For the Rpe activity assay, NADH was purchased from Beyotime Biotechnology (Shanghai, China), thiamine pyrophosphate was purchased from Sangon Biotech (Shanghai, China), triose phosphate isomerase and R5P were purchased from Aladdin Biochemical Technology Co., Shanghai, China, glycerophosphate dehydrogenase was purchased from Shanghai Yuanye Biotechnology Co., Shanghai, China, and Ru5P was purchased from Shanghai ZZBIO Co., Shanghai, China. The authentic riboflavin standard was purchased from Shanghai Macklin Biochemical Co., Shanghai, China.

### Construction of Strains and Plasmids

DNA manipulations were performed using standard protocols. *B. subtilis* transformation was performed according to a published protocol (Anagnostopoulos and Spizizen, 1961). The deletion of *ykgb*, *yqec*, *yqjI*, *gntZ*, and *zwf* was performed using a published method (Liu et al., 2008) based on the paraR-neo/cat-araR counter selection system. The site-direct mutation of *rpe* and other mutations were introduced using the same system with minor modifications. The prinsple of introducing these mutations were also similar with Shi et al. (2013). The *rpe* and *tkt* nonsense mutants (BSRN, BST, BSRT) were constructed by replacing the start codons (ATG) with stop codons (TAA). A schematic of the strategy to construct the mutants is shown in Supplementary Figure 1. Taking BSRN as an example, the primer pairs rpe-ko-UP1/rpe-ko-UP2 and rpe-ko-DN1/rpe-ko-DN2 were used to clone the upstream and downstream sequences flanking the target locus. The primer pair CR1/rpe-ko-CR2 was used to clone the cat-araR fragment, while rpe-ko-CR2 also carried a direct repeat (DR, 20 bp) sequence, the homologous sequence was also located downstream of the UP fragment. The primers rpe-ko-DN1 and rpe-ko-CR2 carried the nonsense mutation of *rpe*, which can be introduced into the downstream and cat-araR fragment. (Other mutations can be carried in upstream fragments as well.) With the nucleotides at the upstream and cat-araR end, the upstream, cat-araR, and downstream fragments can be overlapped to generate the UP-CR-DN fragment. This fragment was used to transform BSLY, after which Cm-resistant transformants were selected, and verified by PCR using the primer pair rpe-ko-UP1/rpe-ko-DN2. The PCR fragment was sequenced to verify the mutations. The verified mutant was incubated in LB broth for 8 h at 37°C to induce intra-genomic recombination at the two homologous DR fragments, after which the culture was plated onto LB agar plates containing Nm. After incubation at 37°C for 1 day, Nm-resistant colonies were selected, and verified by PCR using the primer pair rpe-ko-UP1/rpe-ko-DN2.

The plasmid pMA5(spe) (Dartois et al., 1994), and pHP13(spe) (Peter et al., 1987) in which the Spe^*r*^ replaced the Cm^*r*^ (chloramphenicol resistance) and Ery^*r*^ (erythromycin resistance), were used for the expression of all genes, except for *tktA*. This gene was cloned into the plasmid pET28A (Novagen, Darmstadt, Germany) for Tkt purification. All of the expression plasmids were constructed using Gibson assembly (Gibson et al., 2009). Taking pHP01 as an example, the mutated *rpe* gene was cloned from the genome of BSR (*rpe*^∗^) by PCR using the primer pair rpe1/rpe2. And the PvegI promoter was cloned from the genome of *B. subtilis* 168 by PCR using the primer pair PvegI-1/PvegI-2. The vector was amplified by PCR using primer pair pHP13-1/pHP13-2. The gene, promoter, and vector fragments were assembled using the Clon Expressmulti kit (Vazyme, Nanjing, China). All primers used in their construction are listed in Supplementary Table 2.

### Flask Fermentation Conditions, Measurement of Cell Density, and Riboflavin Titers

To test the riboflavin biosynthesis activity of the engineering strains, 20 μL of glycerol stock of bacteria were mixed with 100 μL of sterile ddH_2_O, spread on an LB agar plate, and incubated at 37°C for 24 h. After that, cells were scraped from the plate and suspended in 1 mL of fermentation medium. The resulting cell suspension was used to inoculate fermentation medium to an initial OD_600_ of 0.1. The fermentation medium contained 40 g/L sucrose, 15 g/L white corn steep powder (Solarbio, Beijing, China), 1.5 g/L corn steep powder (Solarbio, Beijing, China), 5 g/L NH_4_SO_4_, 5 g/L yeast extract (Solarbio, Beijing, China), 0.5 g/L MgSO_4_, 3 g/L K_2_HPO_4_, 1 g/L KH_2_PO_4_, and appropriate antibiotics. The fermentation was conducted at 37°C and 180 rpm for 48 h. Samples were taken at 12, 18, 24, 36, 42, and 48 h to measure the cell growth and riboflavin production. For the strains carrying a nonsense mutation of *tkt* or the pMX45 plasmid, the fermentation time was extended to 60 h. Cell growth was monitored by measuring the optical density (OD) at 600 nm. Determination and calculation of riboflavin concentration was performed as previous paper with some modifications (Himanshi et al., 2012). Culture samples were diluted with 0.1 M NaOH to the linear range of the MAPADA V-1600 spectrophotometer (MAPADA, Shanghai, China). After dissolving for 20 min, 1 mL of the sample was centrifuged at 10,000 × g for 2 min to remove the cells, and other insoluble substances. Then the absorbance at 444 nm was immediately measured. The riboflavin concentration was calculated using a standard equation. All the fermentation experiments were performed in triplicates, and the reported results represented the average of three independent experiments.

### Ribulose 5-Phosphate 3-Epimerase Assays

The method of determining the ribulose-5-phosphate 3-epimerase activity was described in a previous paper (Dante et al., 2003). All enzyme assays were performed at 30°C. The assay principle is based on the consumption of NADH, coupled with a series of enzyme-catalyzed reactions. Before the reaction, the mixtures were equilibrated for 3 min at the reaction temperature, and the reactions were started by the addition of the cell-free extract. The change in the A_340_ due to NADH consumption was measured using a BioTek neo2SM multi-mode reader (BioTek, Vermont, America). The transketolase (TktA from *E. coli*) was purified according to a published method (Amanatuzzakiah et al., 2013). A BCA protein assay kit (Solarbio, Beijing, China) was used to determine the protein concentration. The enzyme activity assays were performed in triplicates, and the reported results represented the average of three independent experiments.

### Analysis of Gene Expression by Quantitative qRT-PCR

Fresh cell culture samples harvested during the exponential growth phase (6 h) in the fermentation medium, were used to extract total RNA using the RNA prep Pure Cell/Bacteria Kit (Tiangen, Beijing, China) following the manufacturer’s instructions. The cDNA was synthesized using the FastQuant RT Kit (Novoprotein, Beijing, China) with gDNase and random primers. The qRT-PCR was carried out on a 7500 Fast Platform (Thermo Fisher Scientific, United States) with Real Master Mix (SYBR Green, Novoprotein, Beijing, China) using 100 ng of cDNA in a total reaction volume of 20 μL with 0.25 mmol/L of each primer (see Supplementary Table 2). The fold change of each transcript in each sample relative to the control was measured in triplicate, normalized to the internal control gene (RNA polimerase, *rpoA*) and calculated according to the comparative CT method (Livak and Schmittgen, 2001).

### Purine Compounds Extraction and HPLC Analysis

Extracellular and intracellular concentrations of purine compounds (adenine, inosine, guanosine, GMP, AMP, IMP, XMP etc.) in the culture suspension were measured using an HP1100 HPLC system (Bio-Rad, United States) equipped with a refractive UV detector. The sampling and measurement was performed according a method described previously (Müller et al., 1996). Before sampling, the tube, containing 0.25 mL 1 N KOH, was placed into liquid nitrogen. Then, during flask fermentation, 9.5 mL of samples were taken at 48 hours, and the samples were frozen in liquid nitrogen, stored at −80°C until analysis. When all of the samples were collected, a further 0.25 mL quantity of KOH was subsequently placed on top of the frozen suspension. The liquid nitrogen-frozen sample was acclimatized to −20°C and then gently thawed at 0–4°C. Then, the samples were centrifuged for 3 min at 20,000 × g at 0–4°C. The supernatant was neutralized with 88% phosphoric acid and analyzed immediately via HPLC. The mobile phase consisted of solvent A [20 mM potassium dihydrogen phosphate, 2.5% acetonitrile, 5 mM tetrabutylammonium hydrogensulfate (TBAHS)] and solvent B (200 mM potassium dihydrogen phosphate, 10% acetonitrile). The analytes were eluted using a linear gradient of 50% solvent A from 0 to 30 min, and 25% solvent A from 30 to 60 min. The measurements were done in three biological replicates which were measured in three technical replicates each, and the reported results represent the average of three independent experiments.

### Growth Assay

Cells were grown aerobically in LB medium overnight, centrifuged at 5,000 × g for 5 min, washed with GMI medium with corresponding carbon source, then adjusted to and OD_600_ of 1.0 and spotted onto GMI plates with 8 g/L glucose/sucrose/maltose/fructose as carbon source, respectively. The plates were incubated for 8 h at 37°C and monitored for growth. The GMI media contained: 2 g/L (NH_4_)_2_SO_4_, 18.3 g/L K_2_HPO_4_, 6 g/L KH_2_PO_4_, 1.2 g/L sodium citrate, 0.4 g/L Casamino acid, 1 g/L yeast extract, 0.2 g/L MgSO_4_⋅H_2_O, and 0.05 g/L tryptophan. The growth of the strains was measured in three technical replicates each.

### Statistical Analyses

All samples were analyzed in triplicate, and the data were presented as the mean ± standard deviation for each sample point. Differences were considered statistically significant if *P*-values lower than 0.05 were obtained.

## Data Availability Statement

The original contributions presented in the study are included in the article/Supplementary Material, further inquiries can be directed to the corresponding author/s.

## Author Contributions

BY designed and performed the fermentations, HPLC analysis, growth test, qRT-PCR analysis, and other analytical experiments. CL developed the idea for the study. YSn constructed the strains, performed the Rpe assay, analyzed the experimental data, drafted, and revised the manuscript. YSu and CL revised the manuscript. SF constructed the plasmids under the guide of MX. All authors read and approved the final manuscript.

## Conflict of Interest

The authors declare that the research was conducted in the absence of any commercial or financial relationships that could be construed as a potential conflict of interest.

## Publisher’s Note

All claims expressed in this article are solely those of the authors and do not necessarily represent those of their affiliated organizations, or those of the publisher, the editors and the reviewers. Any product that may be evaluated in this article, or claim that may be made by its manufacturer, is not guaranteed or endorsed by the publisher.

## Supplementary Material

Supplementary Figure 1 Schematic of the strategy used to construct the mutants investigated in this study. Three steps are needed for the construction of the mutants: PCR, homologous recombination, and intragenomic recombination. Firstly, the UP, CR + DR, and GENE^∗^ + DOWN fragments were cloned by PCR using the primer pairs designated as XXUP-1/XXUP-2, CR1/XXCR-2, and XXDN-1/XXDN-2, where XX indicates the name of the target gene. The primer (as well as the corresponding fragments) XXUP-2 and XXCR-2 carried overlapping sequences with CR and DOWN, respectively, which were used to fuse the sequences by overlap-extension PCR using the primers XXUP-1 and XXDN-2, generating UP(DR)-CR(-DR)-^∗^DN. It should be noted that the first DR (direct repeat, 20 bp), is part of the UP fragment which can be cloned from the genome, and the second DR was introduced by the primer XXCR-2. The mutations were introduced on the primers XXCR-2 and XXDN-1, or alternatively, on XXCR-2 and XXUP-1. Secondly, the overlapped fragment was used to transform *Bacillus subtilis* BSLY, and the transformants were selected on LB plates with 8 μg/mL chloramphenicol. During this process, there was homologous recombination between the fragment and genome via the UP and DOWN fragments. The transformants were verified by PCR using the primers XXUP-1 and XXDN-2, and the mutation was verified by sequencing. Finally, the verified transformants were cultured in LB broth for intragenomic recombination of the DR fragments. The culture was plated onto LB agar plates with 20 μg/mL neomycin. After 1 day, the transformants were selected and verified by PCR using the primers XXUP-1 and XXDN-2. This method is similar to the previously published paraR-neo/cat-araR counter-selection gene deletion system (Liu et al., 2008), and is also similar to another publication (Shi et al., 2013).

Supplementary Figure 2 Comparison of DCW in strains BSR (*rpe*504delA), BSRN (*rpe*^0^), BST (*tkt*^0^), BSRT (*rpe*504delA, Δ*tkt*^0^) and their parent strains in flask fermentation.

Supplementary Table 1 Strains and plasmids used in this study.

Supplementary Table 2 Primers used in this study.

## Abbreviations

PPP: pentose phosphate pathway
EMP: Embden-Meyerhof-Parnas pathway
OPPP: oxidative pentose phosphate pathway
NOPPP: non-oxidative pentose phosphate pathway
Glu: D-Glucose
G6P: 6-O-Phosphono- α-D -glucopyranose
Ru5P: 5-O-Phosphonato-D-ribulose
X5P: 5-O-Phosphono-D-xylulose
R5P: 5-O-Phosphono-D-ribose
E4P: (2R,3R)-2,3-Dihydroxy-4-oxobutyl dihydrogen phosphate
S7P: (2R,3R,4R,5S)-2,3,4,5,7-Pentahydroxy-6-oxoheptyl dihydrogen phosphate
F6P: 6-O-Phosphono- β-D -fructofuranose
PRPP: 1-O-[Hydroxy(phosphonooxy)phosphoryl]-5-O-phosphono- α-D -ribofuranose
IMP: 5 ′ -Inosinic acid
XMP: 5 ′ -2,3-Dihydroxanthylic acid
GMP: 2-Imino-9-(5-O-phosphono- β-D -ribofuranosyl)-3,9-dihydro-2H-purin-6-ol
GTP: Guanosine 5 ′ -(tetrahydrogen triphosphate)
AMP: 5 ′ -Adenylic acid
DARPP: N-(2,5-Diamino-6-oxo-1,6-dihydro-4-pyrimidinyl)-5-O-phosphono- β-D -ribofuranosylamine
ARPP: N-(5-Amino-2,6-dioxo-1,2,3,6-tetrahydro-4-pyrimidinyl)-5-O-phosphono- β-D -ribofuranosylamin
ArPP: 1-[(5-Amino-2,6-dioxo-1,2,3,6-tetrahydro-4-pyrimidinyl)amino]-1-deoxy-5-O-phosphono-D-ribitol
ArP: 1-[(5-Amino-2,6-dioxo-1,2,3,6-tetrahydro-4-pyrimidinyl)amino]-1-deoxy-D-ribitol
DHPB: 2-Hydroxy-3-oxobutyl dihydrogen phosphate
DRL: 1-Deoxy-1-(6,7-dimethyl-2,4-dioxo-3,4-dihydro-8(2H)-pteridinyl)-D-ribitol
FBP: 1,6-Di-O-phosphonato-D-fructose
G3P: 2-Hydroxy-3-oxopropyl dihydrogen phosphate
PYR: 2-Oxopropanoate
G6PD: Glucose-6-phosphate dehydrogenase
6GPD: 6-phosphogluconate dehydrogenase
TBASH: tetrabutylammonium hydrogensulfate
PCR: Polymerase chain reaction
qRT-PCR: real-time quantitative PCR
DR: direct repeat
Nm: neomycin
Cm: chloramphenicol.
