# Supplementary material for: Improving the Production of Riboflavin by Introducing a Mutant Ribulose 5-Phosphate 3-Epimerase Gene in Bacillus subtilis
Source: Front Bioeng Biotechnol. 2021 Jul 29;9:704650. doi: 10.3389/fbioe.2021.704650 (PMC8359813; doi:10.3389/fbioe.2021.704650)
Supplement: Supplementary Figure 1 — Schematic of the strategy used to construct the mutants investigated in this study. Three steps are needed for the construction of the mutants: PCR, homologous recombination, and intragenomic recombination. Firstly, the UP, CR + DR, and GENE∗ + DOWN fragments were cloned by PCR using the primer pairs designated as XXUP-1/XXUP-2, CR1/XXCR-2, and XXDN-1/XXDN-2, where XX indicates the name of the target gene. The primer (as well as the corresponding fragments) XXUP-2 and XXCR-2 carried overlapping sequences with CR and DOWN, respectively, which were used to fuse the sequences by overlap-extension PCR using the primers XXUP-1 and XXDN-2, generating UP(DR)-CR(-DR)-∗DN. It should be noted that the first DR (direct repeat, 20 bp), is part of the UP fragment which can be cloned from the genome, and the second DR was introduced by the primer XXCR-2. The mutations were introduced on the primers XXCR-2 and XXDN-1, or alternatively, on XXCR-2 and XXUP-1. Secondly, the overlapped fragment was used to transform Bacillus subtilis BSLY, and the transformants were selected on LB plates with 8 μg/mL chloramphenicol. During this process, there was homologous recombination between the fragment and genome via the UP and DOWN fragments. The transformants were verified by PCR using the primers XXUP-1 and XXDN-2, and the mutation was verified by sequencing. Finally, the verified transformants were cultured in LB broth for intragenomic recombination of the DR fragments. The culture was plated onto LB agar plates with 20 μg/mL neomycin. After 1 day, the transformants were selected and verified by PCR using the primers XXUP-1 and XXDN-2. This method is similar to the previously published paraR-neo/cat-araR counter-selection gene deletion system (Liu et al., 2008), and is also similar to another publication (Shi et al., 2013). [file Data_Sheet_1.docx]

Supplementary Material

**1. Supplementary Table S1 Bacterial strains and plasmids used in this study**

| Name | Relevant phenotype | Source /reference |
| --- | --- | --- |
| Strains |  |  |
| *B. subtilis* 168 | Wide-type strain, *trpC*2 | Lab stock (from China General Microbiological Culture Collection Center) |
| *E. coli* DH5α | Standard cloning strain | TransGen |
| BSYXM | *B. subtilis* 168, *araR*: para-neo,  ribO^*^,  *rib*+(*gsiB*) | This Study&[27] |
| BSLY | BSYXM, *purA*^*^(P242L) | This Study&[3] |
| BSR | BSLY, *rpe*^*^ (*rpe*504delA) | This Study |
| BSRN | BSLY, Δ*rpe*(*rpe*^0^) | This Study |
| BST | BSLY, Δ*tkt*(*tkt*^0^) | This Study |
| BSRT | BSR, Δ*tkt*(*tkt*^0^) | This Study |
| BSRNE | BSRN/pHP13 (spe) | This Study |
| BSRN1 | BSRN/pHP01 | This Study |
| BSRN2 | BSRN/pHP02 | This Study |
| BSR02 | BSR/pMA5 (spe) | This Study |
| BSR03 | BSR/pMA03 | This Study |
| BSR04 | BSR/pMA04 | This Study |
| BSR05 | BSR/pMA05 | This Study |
| BSR06 | BSR/pMA06 | This Study |
| BSR07 | BSR/pMA07 | This Study |
| BSR08 | BSR/pMA08 | This Study |
| BSR09 | BSR/pMA09 | This Study |
| BSR10 | BSR/pMA10 | This Study |
| BSR21 | BSR, Δ*ykgb* | This Study |
| BSR22 | BSR, Δ*yqec* | This Study |
| BSR23 | BSR, Δ*yqjI* | This Study |
| BSR24 | BSR, Δ*gntZ* | This Study |
| BSR25 | BSR, Δ*zwf* | This Study |
| BSR01 | BSR/pMX45 | This Study |
| BSRT01 | BSRT/pMX45 | This Study |
| BSR02 | BSR01/pHP13 (spe^r^) | This Study |
| BSR03 | BSR01/pHP03 | This Study |
| BSR04 | BSR01/pHP04 | This Study |
| Plasmid |  |  |
| pHP13(spe)  pHP01 | Spe^r^, PvegI  pHP13(spe)-PvegI-*rpe*^*^ | [36]  This Study |
| pHP02 | pHP13(spe)-PvegI-*rpe* | This Study |
| pHP03 | pHP13(spe)-PvegI-*zwf*243 | This Study |
| pHP04 | pHP13(spe)-PvegI-*gnd*361 | This Study |
| pMA5(spe) | Spe^r^, PhpaII | [36] |
| pMA03 | pMA5(spe)-PhpaII-*ribA* | This Study |
| pMA04 | pMA5(spe)-PhpaII-*guaA* | This Study |
| pMA05 | pMA5(spe)-PhpaII-*gmk* | This Study |
| pMA06 | pMA5(spe)-PhpaII-*ykgB* | This Study |
| pMA07 | pMA5(spe)-PhpaII-*prs* | This Study |
| pMA08 | pMA5(spe)-PhpaII-*purF* | This Study |
| pMA09 | pMA5(spe)-PhpaII-*gntZ* | This Study |
| pMA10 | pMA5(spe)-PhpaII-*zwf* | This Study |
| pMX45 | Ery^r^, pSM19035-derived plasmid, carrying a complete *rib* operon | [3] |

**2. Supplemental Table S2 Primers used in this study**

| Name | Sequence (5’–3') |
| --- | --- |
| araRfw | TAGTAAGTAATCGCGTTCAT |
| araR/Para | GCCAGCCCGAGCTCCAATTGACAGAAAATGCAAACAAG |
| Paraf | ATTGGAGCTCGGGCTGGC |
| Nmr | ATTGGAGCTCGGGCTGGC |
| araR/Nmr | TGACCTCTAATAATTGTTAAATTTGAGCCTGAGTTGATCA |
| araRrv | CGGGCAGAGGAATGGGGCTA |
| purA242-UP1 | GATCACTTCAAAGCTAAAGCACA |
| purA242-UP2 | GTTTGTTGAACTAATGGGTGCTTTAGTTGAAGAGATCGTGACACCGCCGGCAACCAGGGTTAGATGACGTAACAAACGGGTATGTTCCTTGGTCGATATCG |
| purA242-CR2 | CCGATCGTGACACCGCCGGCAACCAGGGTTAGATGACGTAACTTATTCATTCAGTTTTCGTGCG |
| purA242-DN1 | GTTACGTCATCTAACCCTGGTTGCCGGCGGTGTCACGATCGG |
| purA242-DN2 | CAACGAGATTTATAATATCACGAAC |
| ribO-UP1 | GAGAGCGAAACAGGACGGTTTTGACC |
| ribO-UP2 | CGTTTGTTGAACTAATGGGTGCTTTAGTTGAAGACCGCCGGTCGGGATTTCCATCCTGCC CCGAAGG |
| ribO-CR2 | GTCACGGGCTCTAAAGCAAATGTGCTTTACTACCGCCGGTCGGGATTTCCATCCTGCCCCGAAGGATACAATTTATTCATTCAGTTTTCGTGCG |
| ribO-DN1 | TAGTAAAGCACATTTGCTTTAGAGC |
| ribO-DN2 | CTTTGTCGGTTTTGCCGTCAGTTTG |
| rpe-mu-UP1 | CTCAGCGGTTCAGCCTTCT |
| rpe-mu-UP2 | TTGAACTAATGGGTGCTTTAGTTGAAGACGCCGCCGTCTACTTCA |
| CR1 | TCTTCAACTAAAGCACCCATT |
| rpe-mu-CR2 | GGAGCGGTTTCTTTGTTGACGCCGCCGTCTACTTCAATTAACAGATCTTTTTTCCTTTTTTATTCATTCAGTTTTCGTGCG |
| rpe-mu-DN1 | TCAACAAAGAAACCGCTCC |
| rpe-mu-DN2 | TCCCCTCCTATAACGTCCC |
| rpe-ko-UP1 | AAGTGCAAATCGCGGTTGA |
| rpe-ko-UP2 | TGAACTAATGGGTGCTTTAGTTGAAGATTATTTATATGCTAATACCTCGGCTTTC |
| rpe-ko-CR2 | GGAAAGAATAGATGGTGCAACCTTTATTTATATGCTAATACCTCGGCTTTCTGTCTTTAATCTTTATTCATTCAGTTTTCGTGCG |
| rpe-ko-DN1 | AGGTTGCACCATCTATTCTTTCC |
| rpe-ko-DN2 | GGCGTTCTTGCTCCGTTATG |
| tkt-ko-UP1 | AGCCAGCGGATATGAAATG |
| tkt-ko-UP2 | TGAACTAATGGGTGCTTTAGTTGAAGAGTGCGAATGGTAGCAACTGAT |
| tkt-ko-CR2 | CTGAGAAGCCGTATTCGTTAATGTGCGAATGGTAGCAACTGATTTCTTTTCAATTGTATCCATTATTCATTCAGTTTTCGTGCG |
| tkt-ko-DN1 | ATTAACGAATACGGCTTCTCAG |
| tkt-ko-DN2 | GCGGGTGATGAATGATTGC |
| ribA1 | TGTACAATAAATGTAGTGAGGTGGATGCAATGTTTCATCCGATAGAAGAAG |
| ribA2 | ACGTTGTAAAACGACGGCCAGTGAATTTAGAAATGAAGTAAATGACCTAGCT |
| purF1 | TGTACAATAAATGTAGTGAGGTGGATGCAATGCTTGCTGAAATCAAAGGCT |
| purF2 | ACGTTGTAAAACGACGGCCAGTGAATTTATTTGGTTAATACTGCTTCTTTTAC |
| prs1 | TGTACAATAAATGTAGTGAGGTGGATGCAATGTCTAAT CAATACGGAGATAAG |
| prs2 | ACGTTGTAAAACGACGGCCAGTGAATTTAGCTGAACAGATAGCTGACTGAT |
| guaA1 | TGTACAATAAATGTAGTGAGGTGGATGCAATGACAAAGTTAGTGAATGAAATGAT |
| guaA2 | ACGTTGTAAAACGACGGCCAGTGAATTTATTCCCACTCAATCGTCG |
| gmk1 | TGTACAATAAATGTAGTGAGGTGGATGCAATGAAAGAAAGAGGGTTATTAA |
| gmk2 | ACGTTGTAAAACGACGGCCAGTGAATTTATTCAACCTCCAGCATTTT |
| ndk1 | TGTACAATAAATGTAGTGAGGTGGATGCAATGATGGAAAAGACTTTTATCATG |
| ndk2 | ACGTTGTAAAACGACGGCCAGTGAATTTAATAGATCCAGCCTGCCA |
| zwf1 | TGTACAATAAATGTAGTGAGGTGGATGCAGTGAAAACAAACCAACAACCAAAA |
| zwf2 | ACGTTGTAAAACGACGGCCAGTGAATTTATATGTTCCACCAGTGTAAGCC |
| gntZ1 | TGTACAATAAATGTAGTGAGGTGGATGCAATGTTCAATTCGATTGGTGTCA |
| gntZ2 | ACGTTGTAAAACGACGGCCAGTGAATTTATTCAGACCAATTCGTATGG |
| ykgB1 | TGTACAATAAATGTAGTGAGGTGGATGCAATGACAAAATACATAGGATAT |
| ykgB2 | ACGTTGTAAAACGACGGCCAGTGAATTTATACTTGATGTAAAAACTTT |
| zwf-ko-UP1 | TATCACCGGAGAACACAACAGATC |
| zwf-ko-UP1 | TGAACTAATGGGTGCTTTAGTTGAAGACTTCCAACTCCTACCACTGCA |
| zwf-ko-CR2 | TCCCAGTGTGCAAAGTTTGTTGCATCGCCTTCCAACTCCTACCACTGCAAACTCTTCTCCGATTTGTCTTATTCATTCAGTTTTCGTGCG |
| zwf-ko-DN1 | GCGATGCAACAAACTTTGCACA |
| zwf-ko-DN2 | AGGTGAGCTTGATAGCTACCTG |
| ygkB-ko-UP1 | CAAGCGGGAACACCCTG |
| ygkB-ko-UP2 | TGAACTAATGGGTGCTTTAGTTGAAGAGAATAAATTCCTTCGCTTCCGCCTTTTGTATATGTTCCCA |
| ygkB-ko-CR2 | GTCAATCGGCCGGTTTCTTTGTCTCGAATAAATTCCTTCGCTTCCGCCTTTTGTATATGTTCCCATTATTCATTCAGTTTTCGTGCG |
| ygkB-ko-DN1 | GAGACAAAGAAACCGGCCG |
| ygkB-ko-DN2 | AGGCGACTGCCTTTGTTAGAG |
| yqjI-ko-UP1 | GGCGTCTGATATGAAAAAACCGC |
| yqjI-ko-UP2 | CGGATGGAATAATAGTCGTGAAAGC |
| yqjI-ko-CR2 | AGCTGTCATAGTAGGCTAATGCGCTTGAGGGTGTTTCCAAGGATTGGACAAATTCTTCAATGCTGTAAGTTATTCATTCAGTTTTCGTGCG |
| yqjI-ko-DN1 | CTCAAGCGCATTAGCCTACTATG |
| yqjI-ko-DN2 | CTTCGCGACATGGTGCAAAAC |
| yqeC-ko-UP1 | GGTTTCTGCTTTTCGCCAATATTGG |
| yqeC-ko-UP2 | TGAACTAATGGGTGCTTTAGTTGAAGACTCAGCCCCGTATGCTTTCAG |
| yqeC-ko-CR2 | GGTTTCCACCGTCCATTTTCCTTCACCCTCAGCCCCGTATGCTTTCAGTTCATCAACAGCCGCTTGATTATTCATTCAGTTTTCGTGCG |
| yqeC-ko-DN1 | GGTGAAGGAAAATGGACGGTG |
| yqeC-ko-DN2 | CCATCGCATAGCCAGGACG |
| gntZ-ko-UP1 | CAAACTGATCGCGGATTCAGG |
| gntZ-ko-UP2 | TGAACTAATGGGTGCTTTAGTTGAAGACCCAGTCCAGCAACGGAAATA |
| gntZ-ko-CR2 | ACTTTTCGCAGGCCTGTTTGATAGGCCCAGTCCAGCAACGGAAATAATGGTTTCCAGCAATGTCCTTATTCATTCAGTTTTCGTGCG |
| gntZ-ko-DN1 | CCTATCAAACAGGCCTGCG |
| gntZ-ko-DN2 | GTTTGGATAACGCCGTCTGG |
| pHP13-1 | ATTCACTGGCCGTCGTTTTACAAC |
| pHP13-2 | AAGCCTGGGGTGCCTAATGAGT |
| PvegI-1 | TTAGGCACCCCAGGCTTGCAAGTTCACCGTCAAGAGTCAATATTC |
| PvegI-2 | TGCATCCACCTCACTACATTTATTG |
| pMA5-1 | CATATGAGTTATGCAGTTTGTAGAATGCAAAAAG |
| pMA5-2 | TAAATCGCTCCTTTTTAGGTGGCACAA |
| rpe-1 | TGTACAATAAATGTAGTGAGGTGGATGCAATGTTTATGATAAAGGTTGCACCATCTATTCTTTC |
| rpe-2 | ACGTTGTAAAACGACGGCCAGTGAATTTATTATTTACTTCCTCTGATTTC |
| rpoA-RT-UP | CGTCGTATCCTCTTATCCTCACTC |
| rpoA-RT-DN | CATCTTCCACAACGCCTTCAAT |
| atpA-RT-UP | CCATCCGATTGAGACGCTTGAG |
| atpA-RT-DN | GTATGCCTGTGCCGCTTTCA |
| tkt-RT-UP | CGTGTATTCGGCGGAACTT |
| tkt-RT-DN | CTGCTGCTGTCTCATTGC |
| gntZ-RT-UP | AAGCATTCAGTGAGCAGCCAAAT |
| gntZ-RT-DN | CCGTCCATATCAGTCCGTTCGTA |
| ywlF-RT-UP | GGCGAAGTTGACAGAGGCATT |
| ywlF-RT-DN | CGATCACCCGTTCACCCATC |
| prs-RT-UP | CGGTGCGACTCGTGTGATT |
| prs-RT-DN | CGATGAGGATAGCAGTCTTCCC |
| zwf-RT-UP | CTGAGAGCACTGCGTCCTATTG |
| zwf-RT-DN | CACCAGCCCATCTGAAGTTGTC |
| ykgB-RT-UP | GACGGTGTGTTGACTGAATCC |
| ykgB-RT-DN | GGATGGCAGAAACGACTTGG |
| yqjI-RT-UP | CTTGACCCAGCTTTAGGCGATT |
| yqjI-RT-DN | GTTCTCCTGATGACAACACA |
| yqeC-RT-UP | CTGATCCTGAGATTGCCAAA |
| yqeC--RT-DN | GCAGTGCGGAAGCATCCT |
| gntZ--RT-UP | CGGTGAAGTCGGTGCGTTAA |
| gntZ-RT-DN | GCATGTCGGCATATTCAA |
| prs-RT-UP | CGGTGCGACTCGTGTGATT |
| prs-RT-DN | CGATGAGGATAGCAGTCTTCCC |
| purE-RT-UP | CTGAACGGAATGGATTCAC |
| purE-RT-DN | CGGGCAAGGTCTTCGTCAAAT |
| guaA-RT-UP | CGACGGCATGACATCTGA |
| guaA-RT-DN | GGCGGCTTACTTGTAATATCA |
| guaB-RT-UP | GCTTCCTCTCGTAGATGACCAGA |
| guaB-RT-DN | CACATCAACATTGGCTTCAACAAG |
| gmk-RT-UP | CAGTAACCACAAGAAGTCC |
| gmk-RT-DN | CCGTAATAATTGCCGACATAC |
| ndk-RT-UP | GGTGTCCAACGTCAGCTCATT |
| ndk-RT-DN | CCTTGATGTTCGGCGTAGT |

## Supplementary Figures


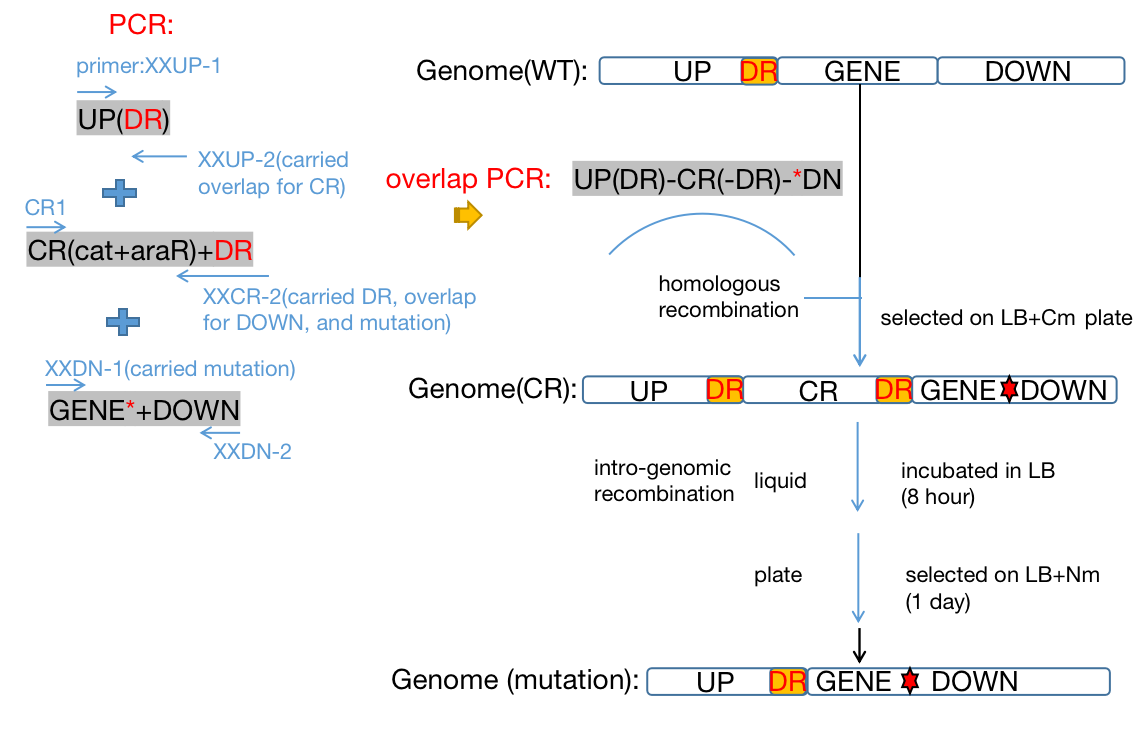


**Supplementary Figure S1. Schematic of the strategy used to construct the mutants investigated in this study.**

Three steps are needed for the construction of the mutants: PCR, homologous recombination, and intragenomic recombination. Firstly, the UP, CR+DR, and GENE*+DOWN fragments were cloned by PCR using the primer pairs designated as XXUP-1/XXUP-2, CR1/XXCR-2, and XXDN-1/XXDN-2, where XX indicates the name of the target gene. The primer (as well as the corresponding fragments) XXUP-2 and XXCR-2 carried overlapping sequences with CR and DOWN, respectively, which were used to fuse the sequences by overlap-extension PCR using the primers XXUP-1 and XXDN-2, generating UP(DR)-CR(-DR)-*DN. It should be noted that the first DR (direct repeat, 20bp), is part of the UP fragment which can be cloned from the genome, and the second DR was introduced by the primer XXCR-2. The mutations were introduced on the primers XXCR-2 and XXDN-1, or alternatively, on XXCR-2 and XXUP-1. Secondly, the overlapped fragment was used to transform Bacillus subtilis BSLY, and the transformants were selected on LB plates with 8 µg/mL chloramphenicol. During this process, there was homologous recombination between the fragment and genome via the UP and DOWN fragments. The transformants were verified by PCR using the primers XXUP-1 and XXDN-2, and the mutation was verified by sequencing. Finally, the verified transformants were cultured in LB broth for intragenomic recombination of the DR fragments. The culture was plated onto LB agar plates with 20 μg/mL neomycin. After 1 day, the transformants were selected and verified by PCR using the primers XXUP-1 and XXDN-2. This method is similar to the previously published paraR-neo/cat-araR counter-selection gene deletion system [39], and is also similar to another publication [40].


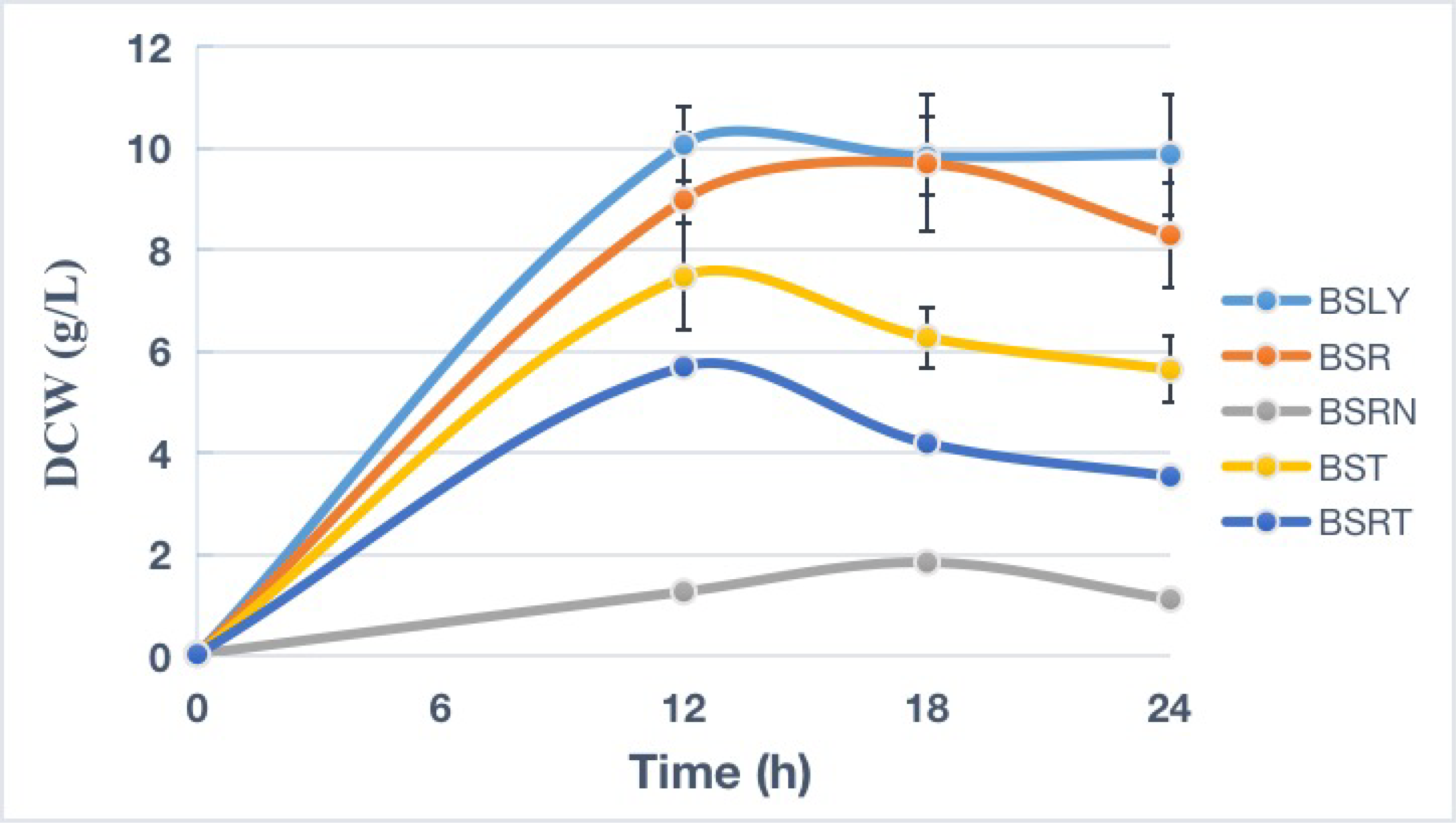


**Supplementary Figure S2 Growth phenotypes the *rpe* and *tkt* mutants**

Comparison of DCW in strains BSR (rpe504delA), BSRN (rpe0), BST (tkt0), BSRT (rpe504delA, Δtkt0) and their parent strains in flask fermentation.
